# Metabolic Needs and Capabilities of *Toxoplasma gondii * through Combined Computational and Experimental Analysis

**DOI:** 10.1371/journal.pcbi.1004261

**Published:** 2015-05-22

**Authors:** Stepan Tymoshenko, Rebecca D. Oppenheim, Rasmus Agren, Jens Nielsen, Dominique Soldati-Favre, Vassily Hatzimanikatis

**Affiliations:** 1 Laboratory of Computational Systems Biotechnology, École Polytechnique Fédérale de Lausanne, EPFL, Lausanne, Switzerland; 2 Department of Microbiology and Molecular Medicine, Faculty of Medicine, University of Geneva, CMU, Geneva, Switzerland; 3 Swiss Institute of Bioinformatics, Quartier Sorge, Batiment Genopode, Lausanne, Switzerland; 4 Department of Chemical and Biological Engineering, Chalmers University of Technology, Gothenburg, Sweden; The Pennsylvania State University, UNITED STATES

## Abstract

*Toxoplasma gondii* is a human pathogen prevalent worldwide that poses a challenging and unmet need for novel treatment of toxoplasmosis. Using a semi-automated reconstruction algorithm, we reconstructed a genome-scale metabolic model, ToxoNet1. The reconstruction process and flux-balance analysis of the model offer a systematic overview of the metabolic capabilities of this parasite. Using ToxoNet1 we have identified significant gaps in the current knowledge of *Toxoplasma* metabolic pathways and have clarified its minimal nutritional requirements for replication. By probing the model via metabolic tasks, we have further defined sets of alternative precursors necessary for parasite growth. Within a human host cell environment, ToxoNet1 predicts a minimal set of 53 enzyme-coding genes and 76 reactions to be essential for parasite replication. Double-gene-essentiality analysis identified 20 pairs of genes for which simultaneous deletion is deleterious. To validate several predictions of ToxoNet1 we have performed experimental analyses of cytosolic acetyl-CoA biosynthesis. ATP-citrate lyase and acetyl-CoA synthase were localised and their corresponding genes disrupted, establishing that each of these enzymes is dispensable for the growth of *T*. *gondii*, however together they make a synthetic lethal pair.

## Author Summary

Understanding the metabolism of disease-causing microorganisms can guide drug design through the identification of metabolic enzymes whose activity is indispensable for important cellular functions. Such understanding can come from the reconstruction and computational analysis of metabolic networks. In this study we have focused on the metabolism of an opportunistic human pathogen, *Toxoplasma gondii*, which chronically infects about 30% of humans worldwide. Through a semi-automatic reconstruction we have developed ToxoNet1, a comprehensive metabolic model of *Toxoplasma gondii*, and we have performed an extensive computational analysis to explore the properties of *Toxoplasma* metabolism. In particular, we have identified and classified the minimal set of substrates the parasite utilizes for growth, along with the genes and pairs of genes that are essential for cellular functions such as growth and energy metabolism. We have validated our computational predictions that the genes encoding acetyl-CoA synthase and ATP-citrate lyase enzymes serve complementary function but their simultaneous disruption does not allow cell growth. This study presents a number of hypotheses generated using ToxoNet1, which can lead to the discovery of novel antiparasitic drug targets.

## Introduction

The phylum of Apicomplexa comprises a large number of obligate intracellular parasites that can infect organisms across the whole animal kingdom. An important member of this phylum, *Toxoplasma gondii*, is a ubiquitous opportunistic pathogen responsible for one of the most common parasitic infections in humans and warm-blooded animals. It is estimated that up to 30% of the human population is chronically infected [1]. Toxoplasmosis is largely asymptomatic in healthy adults but can cause severe disease or even death in immunocompromised individuals and can lead to complications in development of the foetus, if primary infection occurs during pregnancy [2].

*T*. *gondii* possesses a complex life cycle, which is composed of an asexual replicative stage in the intermediate host and a sexually replicative stage within the definitive feline host. During the asexual phase, *T*. *gondii* can switch from a fast-replicative tachyzoite form, which causes acute disease, to a slow-growing bradyzoite stage, which forms cysts that are characteristic of chronic infection. The encysted, slow growing form is resistant to commonly used drugs and immune system attack. Few efficient medicines are available to treat toxoplasmosis and they mainly treat the acute phase of the disease. Furthermore, poor tolerance of these drugs promotes the search for novel drug targets.

Unlike other notorious apicomplexan parasites that infect a narrow range of host cell types, such as the *Plasmodium* species, *T*. *gondii* is able to invade and asexually replicate within virtually any nucleated cell of warm-blooded animals. The broad range of cells amenable to infection by *T*. *gondii* reflects the plasticity of the parasite’s metabolism and versatility in accessing and utilising nutrients to support its intracellular growth [3,4]. The complexity of decoupling the metabolic processes of this intracellular pathogen from those of the infected host limits the depth of our understanding about the metabolic capabilities of *T*. *gondii*.

Currently, no adequate experimental approaches exist to answer comprehensively the following important questions: 1) what substrates are available within the host cell that are necessary for *T*. *gondii* replication and which of these are dispensable; 2) in which intracellular compartments do the enzymatic activities annotated at the genome level occur; 3) which of these enzymatic activities are indispensable for replication or other vital processes of the parasite. While achievable, the application of high-throughput gene knockout or knockdown strategies to globally determine gene essentiality in *T*. *gondii* remains a major undertaking.

Computational (i.e. *in silico*) metabolic modelling coupled with systematic analyses facilitates the study of biological systems. This modern approach of systems biology has been extensively exerted to predict gene essentiality in various bacteria, including numerous pathogenic species [5]. *In silico* metabolic models offer a cost-effective pipeline to identify putatively indispensable metabolic processes that, in the case of pathogens, represent potential targets for therapeutic intervention [6]. Recently, models have been constructed for eukaryotic pathogens including for members of the phylum Apicomplexa [7–10].

In this study we have reconstructed a genome-scale metabolic model of *T*. *gondii*, ToxoNet1, aiming to address the abovementioned questions in a systematic way and to the extent possible with the currently available knowledge. Using flux-balance analysis we have identified genes, reactions and pairs of genes for which deletion renders production of biomass components impossible. Furthermore, we have assessed which precursors are necessary for each of the biomass components and have defined minimal sets of such precursors that allow *in silico* simulation of growth. To illustrate the applicability of ToxoNet1 in filling knowledge gaps regarding parasite metabolism, we experimentally challenged the model prediction regarding the alternative routes for generation of cytosolic acetyl-CoA. We have confirmed *in vitro* the functional redundancy and synthetic lethality of the two biosynthetic routes that involve the ATP-citrate lyase and acetyl-CoA synthase enzymes.

## Results

**Fig 1:**
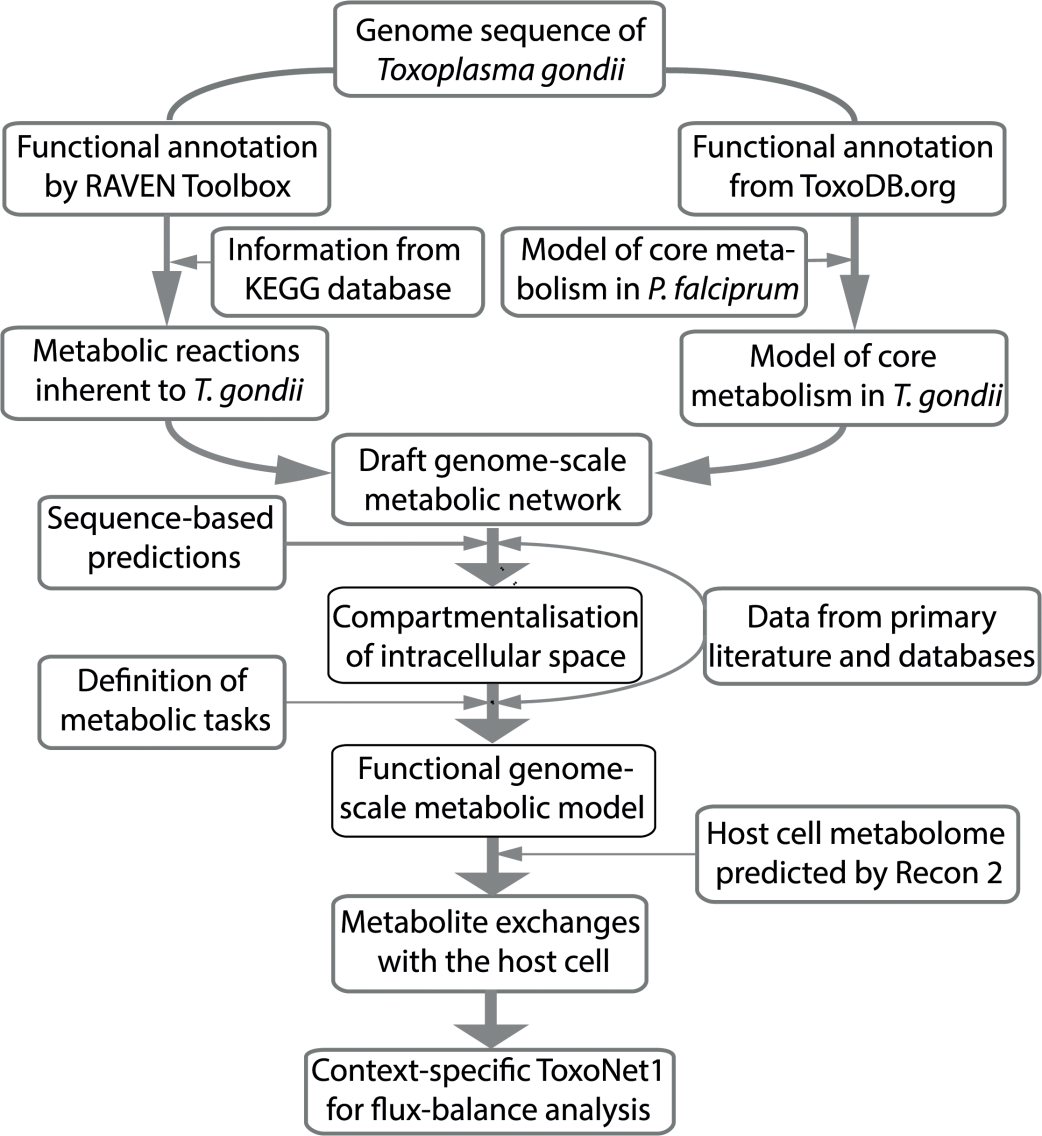
Reconstruction workflow for ToxoNet1 summarized as a flowchart illustration.

Here we present the full genome-scale *in silico* reconstruction of metabolism in *T*. *gondii* with manually refined gene-reaction associations—ToxoNet1. The model reconstruction process required completion of the following major steps (schematically shown in Fig 1): (1) reconstruction of the draft metabolic network; (2) compartmentalization of the intracellular space; (3) verification of the metabolic capabilities and manual literature-based corrections; (4) representation of the plausible exchanges of metabolites between the infected host cell and the parasite. In general terms, the reconstruction process was consistent with the workflow that has been previously used for semi-automated reconstruction of a genome-scale metabolic model for *Penicillium chrysogenum* by means of the RAVEN Toolbox [11]. All the necessary manual corrections were made in accordance to the conventional model reconstruction protocol [12].

For the compartmentalization process we consulted the models of *Plasmodium falciparum* [9,13] and used sequence-based predictors of subcellular localization [14–16] as well as the ApiLoc (http://apiloc.biochem.unimelb.edu.au/apiloc/apiloc) database. The databases used for identification of “gap” reactions were KEGG [17] and LLAMP [18]. Recon 2 [19] was used as a model of host cell metabolism to define the list of putatively host-supplied substrates.

### Reconstruction of the ToxoNet1

Reconstruction of the metabolic network was achieved by combination of the state-of-the-art algorithm for a semi-automated generation of metabolic networks [11] and a comprehensive manual curation based on the relevant primary literature. Details of the reconstruction process are provided in the materials and methods section and the most important steps are discussed below.

The reconstructed metabolic network accounted for 527 open-reading frames (ORFs) that were linked to 867 unique metabolic reactions present in the KEGG [17] database. Each functional annotation in the model was assigned with two estimates (namely a bit-score and e-value), which indicated confidence of association for a given ORF with a corresponding enzymatic function.

**Fig 2:**
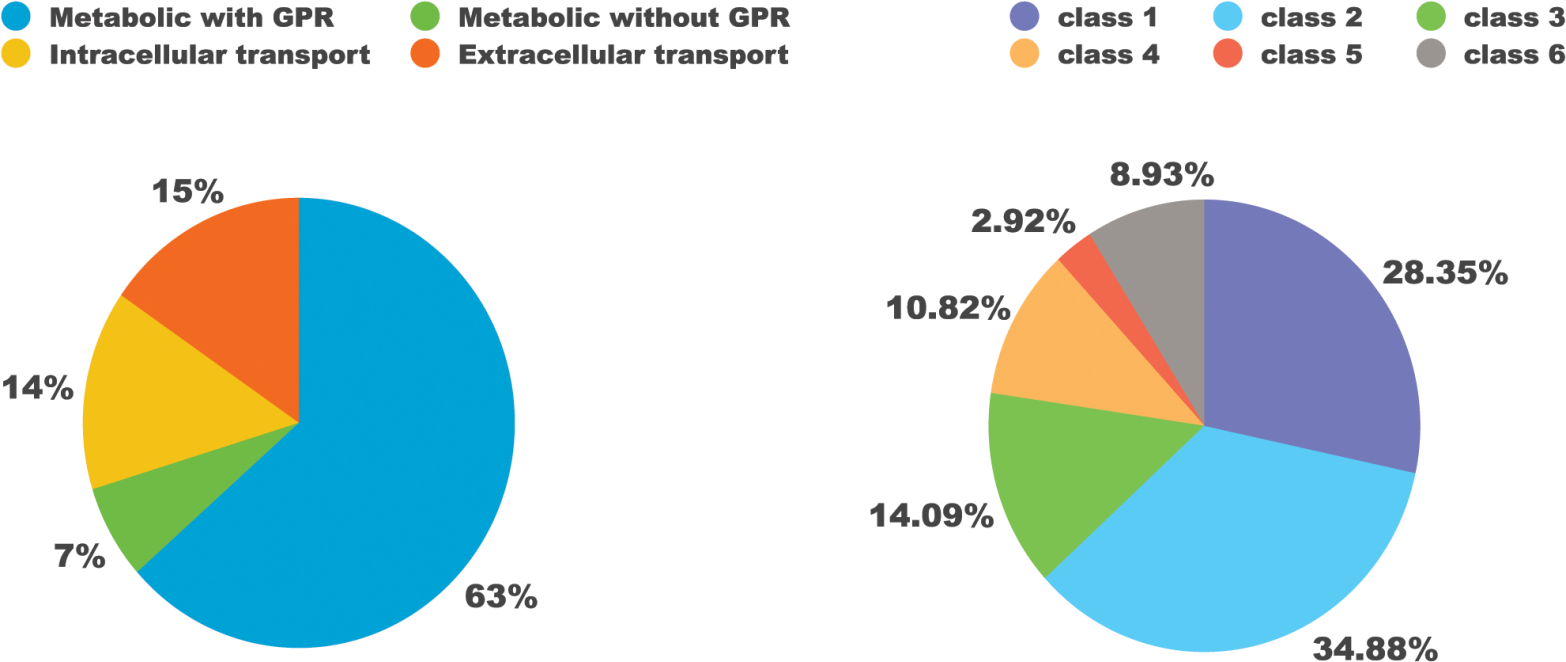
Breakdown of the metabolic network. A) By presence of gene-reactions association; B) By enzyme classes encoded in the genome of *T*. *gondii* (EC nomenclature, class 1: oxidoreductases, class 2: transferases, class 3: hydrolases, class 4: lyases, class 5: isomerases, class 6: ligases).

In ToxoNet1, the majority of reactions were associated with genes that encode corresponding metabolic enzymes (Fig 2A). Intracellular metabolic reactions not associated with any genes comprised only 9.6% of all the metabolic reactions present in the model (6.7% of all the reactions in the model); they include spontaneous reactions (not enzyme-catalysed) and so-called gap-filling reactions that were added for correct functioning of the model. Most of the metabolite transport reactions, which were added to connect pathways segregated between subcellular compartments, also lacked known gene associations.

Among the metabolic enzymes encoded in the genome of *T*. *gondii* and present within ToxoNet1, the transferases (class 2) and oxidoreductases (class 1) were the most numerous (over 60% of all the enzymes). Hydrolases (class 3), lyases (class 4) and ligases were significantly less represented (Fig 2B). The least frequent were isomerases (class 5), which is in accordance with the models of other pathogens, such as *Leishmania major* [20] and *Plasmodium falciparum* [8].

### Compartmentalization of the ToxoNet1

Relatively straightforward experimental methods exist to determine the localization of proteins in *T*. *gondii* [21], however, it would be laborious and expensive to apply such methods for hundreds of enzymes. According to the ApiLoc database (http://apiloc.biochem.unimelb.edu.au/apiloc/apiloc), experimental data was available only for a limited number of proteins: 60 out of the 527 included in ToxoNet1. Thus the only reasonable option for building a compartmentalized metabolic model was to make use of sequence-based localization predictors. To define putative localizations of the enzymes with no experimental evidence we generated sequence-based predictions using three software algorithms: TargetP [16], MitoProt II [15], and ApicoAP [14]. We then manually reconciled output data of the three independent predicting algorithms and assigned the localization based on the manually determined consensus prediction, also considering recent primary literature whenever it was available (S1 Table).

Comparison of these predictions with 60 experimentally established subcellular localizations available from ApiLoc uncovered two issues: (i) not all the computational predictions matched their experimental data (highlighted in the S1 Table); (ii) some of the enzymes were reported to be present in two and, in one case (glutathione/thioredoxin peroxidase [22]), three different subcellular compartments defined in ToxoNet1. In these cases the sequence-based predictors were not efficient and suggested only one of the compartments. Therefore, we assigned compartments in a supervised manner considering all available evidence.

The information on compartmentalisation organised according to the global metabolic subsystems of ToxoNet1 is shown in the Table 1.

**Table 1:** Breakdown of ToxoNet1 by metabolic subsystems and subcellular compartments.

Metabolic subsystems	Genes	Reactions	Reactions by compartments (non-unique)		
		(unique)	cytosol	mitochondrion	apicoplast
Carbohydrates	88	114	101	32	16
Amino acids	105	163	140	56	8
Nucleic acids	74	105	93	8	19
Fatty acids	50	99	59	41	45
Vitamins, Co-factors	78	114	111	29	22
Phospholipids	34	42	44	3	2
Miscellaneous	98	230	194	38	28
Total	527	867	742	207	140

In ToxoNet1 a majority of the reactions (c.a. 69%) occur in the cytosolic compartment. We also considered this compartment as a default for enzymes with a subcellular localization remaining unclear from the *in silico* predictions. The mitochondrion accommodates 19% of all metabolic reactions of the model. The remaining 12% are localized to the apicoplast, a plastid-like non-photosynthetic organelle [23]. We connected these two compartments to the cytosol by 223 transport reactions. This enabled the corresponding number of metabolites to be transported across these boundaries, which delineate the organellar membranes (for further details see the materials and methods section). We allowed the metabolites present in the mitochondrion or apicoplast to be reversibly transported to and from the cytosol if they satisfied the following requirements: (i) metabolite has to be present (i.e. participate in at least one reaction) in both the cytosol and corresponding non-cytosolic compartment; (ii) it should be neither phosphorylated nor containing an acyl-carrier-protein or CoA moiety, unless supporting evidence of such transport is available. We also allowed: (i) import of phosphoenolpyruvate and dihydroxyacetone phosphate from the cytosol to the apicoplast [24]; (ii) export of ATP from the mitochondrion to the cytosol (TGME49_249900); (iii) export of isopentenyl pyrophosphate (IPP) and dimethylallyl pyrophosphate (DMAPP) from the apicoplast to the cytosol. The latter allowed us to observe an unusual feature of the isoprenoid biosynthesis pathway in *T*. *gondii* regarding the isopentenyl pyrophosphate isomerase (EC 5.3.3.2). This enzyme interconverts IPP into its more reactive isomer—DMAPP. In *T*. *gondii*, as well as in *Plasmodium spp*. this enzyme is absent [25]. Thus, both IPP and DMAPP have to be produced and transported from the apicoplast as separate entities for synthesis of long isoprenoid chains in the cytosol.

### Verification of the metabolic capabilities of ToxoNet1

A major limitation of all algorithms for automatic reconstruction of metabolic models is the number of missing reactions (so-called gaps) that disrupt metabolic pathways. The RAVEN Toolbox offers a functionality called metabolic tasks (*checkTasks and fitTask* functions), which verifies that specific tasks are fulfilled and, if necessary, it fills the gaps in the metabolic pathways to meet the required functionality.

We created metabolic tasks for the synthesis of every metabolite included in the biomass reaction (complete formulation of all the tasks is provided in the S2 Table). The outcomes of the tasks were categorized in three groups shown in Table 2. The first group contains the metabolites that could be produced from glucose and inorganic compounds (i.e. *de novo* synthesis was possible in the model). The second group contains the metabolites whose synthesis required a precursor with a specific moiety (e.g. hypoxanthine or other source of purine moiety for purine nucleotides and their derivatives). The third group are the biomass constituents that could not be produced even with the maximum set of host-supplied substrates, and therefore they were directly taken up from the host (e.g. choline, arachidonic acid, cholesterol).

**Table 2:** Nutritional requirements of *T*. *gondii* predicted *in silico*.

	Produced *de novo* (from glucose and inorganic substrates)	Production requires specific precursors (secondary production)	No production from any of the available precursors
**Amino acids**	Alanine; Asparagine; Aspartate; Cysteine; Glutamate; Glutamine; Proline; Serine; Threonine	Tyrosine^1^; Glycine^2^; Lysine^3^; Valine^4^; Leucine^5^; Isoleucine^6^; Methionine^7^ ^,^ ^8^; Phenylalanine^8^	Arginine; Histidine; Tryptophan
**Nucleotides**	dCTP; dTTP; CTP; UTP	dATP^9^; dGTP^9^; ATP^9^; GTP^9^	
**Fatty acids**	Dodecanoate; Tetradecanoate; Octadecanoate; Hexadecanoate; Octadecenoate		Linoleate; Arachidonate
**Cofactors**	Pyridoxal phosphate; Protein-N^6^-(lipoyl)lysine[apicoplast]^15^	Glutathione^2^ ^,^ ^9^; CoA^9^ ^,^ ^10^; FMN^11^; NAD^9^ ^,^ ^12^ NADP^9^ ^,^ ^12^; Thiamin diphosphate^13^; S-Adenosyl-L-methionine^2^ ^,^ ^7^ ^,^ ^9^; Protein N6-(lipoyl)lysine [mitochondrial]^16^; Tetrahydrofolate^2^; Tetrahydrobiopterin^14^	
**Other membrane precursors**	UDP-N-acetyl D-glucosamine; Geranylgeranyl-PP; Undecaprenyl-PP; PhosphatidylethanolaminePhosphatidylserine	Phosphatidylcholine^17^; Sphingomyelin^17^; ADP-glucose^3^ GDP-mannose^2^; 1-Phosphatidyl-D-myo-inositol^18^	Cholesterol

Required precursors:

^1^—phenylalanine or phenylpyruvate;

^2^—folic acid or its derivatives;

^3^—l-2-aminoadipate 6-semialdehyde;

^4^–3-methyl-2-oxobutanoic acid;

^5^–4-methyl-2-oxopentanoate;

^6^ –(s)-3-methyl-2-oxopentanoic acid;

^7^—l-homocysteine;

^8^—phenylpyruvate;

^9^—a source of purine moiety: hypoxanthine, adenine, adenosine or similar;

^10^—pantothenate or valine+β-alanine;

^11^—riboflavin;

^12^—nicotinic acid or its derivatives;

^13^—thiamine;

^14^–4-hydroxybenzoate;

^15^—an undefined”sulfur donor” molecule;

^16^—lipoic acid;

^17^—choline;

^18^—myo-inositol.

According to ToxoNet1, *T*. *gondii* has the capability to produce *de novo* a number of biomass precursors such as pyrimidine nucleotides and their derivatives, fatty acids, isoprenoids and about half of the proteinogenic amino acids (Table 2). When tasks of *de novo* production for certain biomass constituents failed we attempted to produce them by supplying additional precursors. In some cases uptake of one or more molecule(s) from the list of host-supplied molecules, enabled production of the necessary biomass precursors in ToxoNet1. Nevertheless, several biomass building blocks could not be produced in the model even when all the 230 host-supplied substrates were provided simultaneously, indicating that the parasite is likely auxotrophic for these molecules.

In the cases when metabolic tasks could not be accomplished despite literature evidence (mainly acquired from the LLAMP database [18]), we performed gap-filling. This is a conventional part of building genome-scale metabolic models [12] and it implies inclusion of a minimal number of reactions to complete pathways of interest. We used both the automated gap-filling function of the RAVEN Toolbox (*fillGaps*) and manual gap-filling based on the information from the LLAMP database [18]. For instance, the threonine biosynthesis pathway in ToxoNet1 was nearly complete but lacked a single enzyme, the homoserine dehydrogenase (E.C. 1.1.1.3). The corresponding reaction (R01773) was included in the model without a gene assigned (i.e. as a “gap-fill”) justified by the presence of four other enzymes in the pathway and the fact that the immediate downstream enzyme was identified in a proteomics study [26]. ToxoNet1 also retrieved a known issue of the lysine biosynthesis pathway: the bacterial-type pathway consisting of 9 enzymatic steps lacks enzymes annotated for 4 sequential steps, making the presence of the functional pathway debatable. While this issue remains unresolved, we decided not to gap-fill this pathway in the model, thus, leaving lysine an essential amino acid. The full list of gap-fill reactions included in ToxoNet1 is provided in S3 Table.

Importantly, some of the information necessary for a metabolic model could not be easily deduced from the genome sequences alone. This is why we merged the draft output metabolic network from the RAVEN Toolbox with a small-scale model of central carbon metabolism in *T*. *gondii*. We manually built such a model prior to this study based on the genome annotation from ToxoDB and refined it according to the relevant primary literature. This model contained a number of recent experimental findings as well as manually assembled complex gene-reaction associations. Among these were the pyruvate dehydrogenase activity that can be carried out by the branched-chain keto-acid dehydrogenase complex (BCKDH) in the mitochondrion [27], mitochondrial pyruvate transporter [28–30] and the GABA-shunt connected to the tricarboxylic acid (TCA) cycle [3].

### Substrate dispensability based on ToxoNet1

One of the major challenges in simulation of parasite metabolism *in silico* is the unknown range of metabolites available for the parasite within the host cell. As extracellular replication (axenic cultivation) of *T*. *gondii* is not possible, it remains undefined which of the substrates are dispensable for the parasite and which are not.

To address this issue *in silico*, we developed a method to enumerate all of the smallest/ minimal metabolite sets (further referred to as *in silico* minimal medium or IMM), which could enable simulation of growth in ToxoNet1. In brief, the algorithm applied iterative rounds of biomass production using the fewest number of substrates possible. After each of the iterations we added a constraint to ensure that the next IMM included at least one substrate uncommon from the preceding ones (further details on the formulation of the algorithm are described in the materials and methods section).

The results of these simulations showed that as few as 19 substrates were sufficient for simulation of *T*. *gondii* replication in ToxoNet1. We also observed a relatively large number (2592) of alternative sets of 19 substrates with 10 metabolites being constitutively present in all the IMMs. The other 9 substrates could be substituted by at least one other host-supplied metabolite (Table 3). Despite the very large theoretical number of combinations (in the case of 9 variable substrates picked from 220) we observed only 2592 alternative IMM of 19 substrates. This indicates the ability of ToxoNet1 to substitute one substrate with another from the set of 230 is rather limited. Indeed, the majority of the 2592 IMMs arise from flexibility in the carbon source (one out of 7 available) and a source of nicotinate moiety (one out of 3). The other metabolites are either non-substitutable (10) or substitutable with one single alternative. However, in this particular analysis, we excluded the possibility of substituting one metabolite with simultaneous uptakes of several others, as it would lead to more than the minimal number of substrates used.

**Table 3:** Composition of the minimal *in silico* media that allows ToxoNet1 to simulate replication of *T*. *gondii*.

**Indispensable** (biomass precursor for which it is necessary)	
5,10-methyltetrahydrofolate (THF); cholesterol (itself); choline (phosphatidylcholine); L-arginine (proteins); L-histidine (proteins); L-tryptophan (proteins); lipoate (lipoylation in mitochondrion); riboflavin (FAD); S-adenosyl-L-homocysteine (purine nucleotides, methionine, SAM); thiamine (TPP)	
**Dispensable** (multiple alternative precursors present in different minimal media sets)	
C6 or C5 carbon source	D-fructose or D-glucosamine or D-glucose or D-mannose or D-ribose or D-sorbitol or 2-Deoxy-D-ribose
NAD/NADP precursors	nicotinate or nicotinate-D-ribonucleoside or nicotinamide
Amino acids or precursors	L-lysine or L-2-aminoadipate 6-semialdehyde
	L-isoleucine or (S)-3-methyl-2-oxopentanoate
	L-valine or 3-methyl-2-oxobutanoate
	L-leucine or 4-methyl-2-oxo-pentanoate
	L-phenylalanine or phenylpyruvate
Source of inorganic iron	Fe^2+^ or heme
Source of inorganic phosphate	orthophosphate or diphosphate

Abbreviations: FAD—flavine-adenine dinucleotide, NAD—nicoticamide-adenine dinucleotide, NADP—NAD phosphate, TPP—thiamine pyrophosphate, SAM—S-adenosyl-methionine, THF—tetrahydrofolate.

### Gene essentiality predicted by ToxoNet1

We simulated *in silico* the outcomes of systematic removal of genes and reactions in ToxoNet1 to explore which of them represent indispensable metabolic functions. With the full set of 230 host-supplied substrates out of the 527 genes in ToxoNet1, 53 genes were predicted to be essential (Table 4 and literature evidence [31–38]). Considering that most of the transport reactions do not have known gene-reaction associations, we also simulated single reaction deletions. This allowed us to assess the dispensability of metabolite transport across the compartments, as well as metabolite exchanges between *T*. *gondii* and its host cell (Table 4 and S5 Table).

In addition to single gene and reaction essentiality, we also simulated double gene deletions to reveal the pairs in which genes are not essential for replication on their own, yet deleterious when disrupted together. A total of 20 pairs that caused such synergistic effect (synthetic lethality) are listed in S5 Table.

**Table 4:** Gene essentiality predictions of ToxoNet 1 and available literature evidence.

Gene identifiers	Metabolic subsystem	E.C. identifier	Essentiality of ortholog(s) in *P*. falciparum*	Essentiality evidence
TGME49_264780	Amino sugar and nucleotide sugar metabolism	2.7.7.23	no	NA
TGME49_264650		5.4.2.3		NA
TGME49_309730	Antioxidative metabolism	1.8.1.7	yes	NA
TGME49_298990	Biosynthesis of terpenoids	1.18.1.2	no	Inhibition of the pathway [31],[32]
TGME49_316770		2.5.1.-		NA
TGME49_269430		2.5.1.1	yes	Inhibition of the pathway [31],[32]
TGME49_227420		1.17.1.2		
TGME49_306260		2.7.7.60		
TGME49_255690		4.6.1.12		
TGME49_306550		2.7.1.148		
TGME49_262430		1.17.7.1		
TGME49_214850		1.1.1.267		
TGME49_208820		2.2.1.7		
TGME49_224490		2.5.1.1 2.5.1.10		
TGME49_290600	Citrate cycle(TCA cycle)	6.2.1.5	no	Growth reducing knockout [33]
TGME49_278910	Cysteine and methionine metabolism	2.5.1.47	no orthologs	NA
TGME49_225990	Fatty acid biosynthesis (apicoplast)	2.3.1.39	no (not essential on blood- and essential in liver stage)	Conditional knockout of apicoplast-targeted acyl-carrier protein [34]
TGME49_217740		1.1.1.100		
TGME49_206610		2.3.1.12		
TGME49_231890		2.3.1.180	yes	
TGME49_251930		1.3.1.9		
TGME49_321570		4.2.1.-		
TGME49_293590		2.3.1.179		
TGME49_221320		6.4.1.2 6.3.4.14		
TGME49_239710	Fructose and mannose metabolism	5.4.2.8	no	NA
TGME49_239250	Glycerolipid metabolism	2.7.1.107	no orthologs	NA
TGME49_310280	Glycerophospholipid	2.7.7.14	no	NA
TGME49_261480	metabolism	2.7.8.-		Growth arrest upon inhibition [35]
TGME49_212130		3.1.1.3 2.3.1.-	no orthologs	NA
TGME49_216930		2.7.7.15	yes	Growth reducing knockout res-cued by salvage from host [36]
TGME49_281980		2.7.7.41		NA
TGME49_233500	Glycolysis / Gluconeogenesis	5.3.1.1	yes	NA
TGME49_207710	Inositol phosphate metabolism	2.7.8.11	yes	NA
TGME49_315640	Lipoic acid biosynthesis	2.3.1.181	no	Conditional knockout of apicoplast-targeted acyl-carrier protein [34]
TGME49_226400		2.8.1.8		
TGME49_271820	Lipoic acid metabolism in mitochondrion	2.7.7.63	no	NA
TGME49_269800	Nicotinate and nicotinamide metabolism	6.3.5.1	yes	NA
TGME49_244700		2.7.1.23		
TGME49_224900	Purine metabolism	2.7.4.3	no	NA
TGME49_233110		1.1.1.205	yes	NA
TGME49_242730		2.7.4.8		
TGME49_230450		6.3.5.2		
TGME49_257740	Pyrimidine metabolism	2.7.4.- 2.7.4.14	no	NA
TGME49_249180		1.5.1.3 2.1.1.45	yes	[37]
TGME49_306970		2.7.4.9		NA
TGME49_299210		6.3.4.2		NA
TGME49_305980	Pyruvate metabolism in apicoplast	1.8.1.4	no^**^	NA
TGME49_299070		2.7.1.40	yes	NA
TGME49_216740	Riboflavin metabolism	2.7.1.26	yes	NA
TGME49_214280		2.7.7.2		
TGME49_237200	Sphingolipid metabolism	1.14.-.-	no orthologs	NA
TGME49_316450		2.3.1.24	yes	
TGME49_215250	Thiamine metabolism	2.7.6.2	yes	NA

(NA denotes the cases when neither supporting nor contradicting literature reference could be found). Gene essentiality in *P*. *falciparum* is based on the Supplementary Table 1 of the review manuscript [38].

Gene essentiality predictions depend on the following important aspects of metabolism as represented in the model: (1) range of substrates that can enter the model (i.e. molecules that the parasite can take up from the infected host cell); (2) composition of the parasite cell represented as a set of biomass precursor molecules (the concept of biomass objective functions is explained in [39]); (3) the presence of alternative metabolic routes to produce biomass building blocks from the different substrates. Assuming a very permissive range of 230 substrates as potentially accessible for the parasite we predicted a minimal set of essential genes and reactions. In consequence, the number of genes predicted by ToxoNet1 as non-essential is likely to be overestimated. Yet with this assumption the probability of incorrect prediction of genes to be essential is lower.

### ToxoNet1 as a framework for *in silico* assessment of experimental research questions

**Fig 3:**
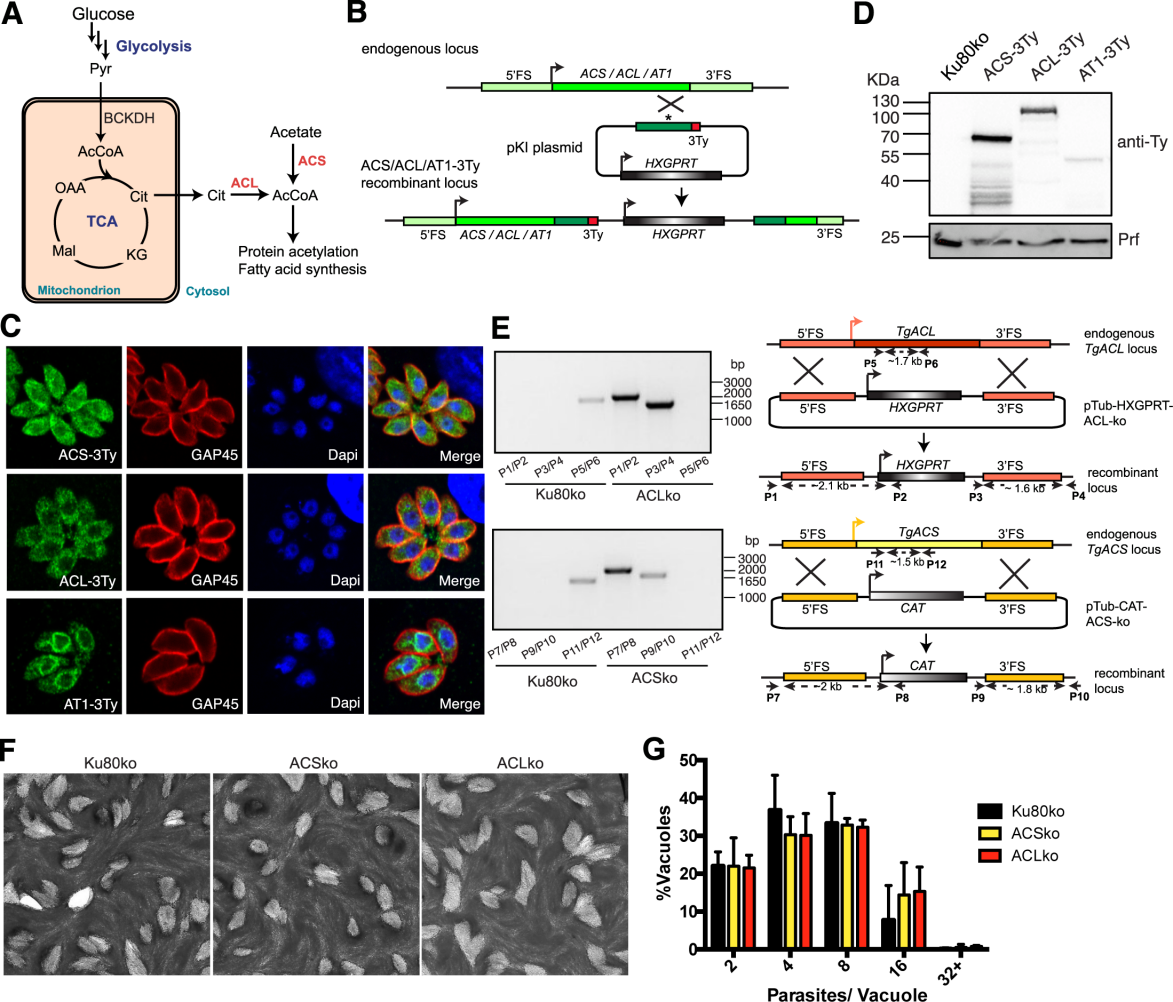
Both ACS and ACL are dispensable in the tachyzoite stage of *T*. *gondii*. (A) Schematic representation of the two pathways to produce acetyl-CoA in the cytosol of *T*. *gondii*. Abbreviations: AcCoA, acetyl-CoA; α-KG, α-ketoglutarate; Cit, citrate; Glc, glucose; Lac, lactate; Mal, malate; OAA, oxaloacetic acid; Pyr, pyruvate; Suc, succinate. Enzymes in red: ACL, ATP-citrate lyase; ACS, Acetyl-CoA synthetase. (B) Scheme of the knock-in strategy used to introduce a 3Ty-tag in the endogenous loci of ACS, ACL and AT1. (C) Localization of endogenous ACS, ACL and AT1 C-terminally Ty-tagged (ACS-3Ty, ACL-3Ty and AT1-3Ty) in the cytoplasm, cytosol and endoplasmic reticulum respectively of intracellular parasites using anti-Ty as well as anti-GAP45 that stains the periphery and DAPI which stains the nucleus of the parasite. (D) Immuno-blot of total lysates from Ku80ko parasites expressing the C-terminally Ty-tagged endogenous ACS, ACL and AT1 proteins by Western blot using anti-Ty antibodies. Anti-Profilin (Prf) represents a loading control. (E) Schematic representation of the direct knockout strategy by double homologous recombination where ACS was replaced by the chloramphenicol resistance cassette and ACL by the HXGPRT selection cassette. The position of the primers used to confirm the integration and the length of the PCR products are indicated. PCRs performed on genomic DNA extracted from Ku80ko, ACSko and ACLko strains confirm the integration of the selection cassette and loss of the corresponding gene locus. The sequences of the primers can be found in S7 Table. (F) Plaque assays performed with Ku80ko, ACSko and ACLko parasite lines fixed after 7 days. No significant defect in the lytic cycle could be observed. (G) Intracellular growth assay performed on Ku80ko, ACSko and ACLko strains by determining the number of parasites per vacuole 24h post infection. Data are represented as mean ± SD from 3 biological replicates.

In order to evaluate whether ToxoNet1 predictions closely reflect the metabolic capabilities of *T*. *gondii* observed experimentally in tissue culture, we chose to assess the importance of the two independent routes of cytosolic acetyl-CoA production in the rapidly dividing tachyzoite stage (Fig 3A). Acetyl-CoA is an important molecule in central carbon metabolism that is involved in many biochemical processes such as fatty acid synthesis (FAS type I pathway), fatty acid chain elongation and acetylation of proteins, in particular histones. ToxoNet1 identified two enzymes that can produce acetyl-CoA in the cytosol: (1) from acetate through the acetyl-CoA synthase (ACS) reaction (TGME49_266640), (2) from TCA-derived citrate by ATP-citrate lyase (ACL) (TGME49_223840), as shown in Fig 3A. Acetoacetate-CoA ligase (TGME49_219230) produces acetoacetyl-CoA that can be converted to acetyl-CoA by the acetyl-CoA acetyltransferase (TGME49_301120), which belongs to the fatty acid degradation pathway in mitochondrion. We thus concluded that this is unlikely to be producing cytosolic acetyl-CoA production due to the predicted mitochondrial localization of the two enzymes. In further support of the importance of cytosolic acetyl-CoA production is the presence of a putative ortholog of the human acetyl-CoA transporter (AT1) in the *T*. *gondii* genome [40]. This transporter was shown to localize at the endoplasmic reticulum (ER) membrane and to be essential for the survival of eukaryotic cells by allowing import of acetyl-CoA from the cytosol to the ER to acetylate proteins within this compartment. ToxoNet1 predicted ACS- and ACL-encoding genes to be fully dispensable when knocked out individually, however, their simultaneous knockout was predicted to be lethal (S5 Table).

### Experimental investigation of acetyl-CoA biosynthesis in the cytosol and its essentiality

To determine the localization and level of expression of ACS and ACL in *T*. *gondii*, we modified the endogenous locus (knock-in) by introducing a 3xTy-epitope tag at the C-terminal end of both genes in the RH*ku80ko* (Ku80ko) background strain, which limits random integration in the genome hence facilitating recovery of homologous recombination events (Fig 3B). ACS is clearly cytosolic and nuclear whereas ACL appears to localize mostly to the cytosol, whilst a fainter nuclear staining can also be detected by indirect immunofluorescence assay (IFA) (Fig 3C). Localization of the acetyl-CoA transporter AT1 in the perinuclear region of the ER in *T*. *gondii* was validated using the same knock-in tagging strategy (Fig 3B and 3C). Expression of the epitope-tagged proteins was further validated by Western blot analyses, where ACS runs at the expected molecular weight of ~80 kDa, ACL at ~140 kDa and AT1 at ~65 KDa (Fig 3D). Comparative signal intensity observed on Western blots suggests that ACS is significantly more abundant than ACL and AT1.

To functionally assess the importance of both routes to produce cytosolic acetyl-CoA and challenge the predictions made by ToxoNet1, individual deletion of the genes encoding ACL and ACS were achieved using a double homologous recombination strategy (ACLko and ACSko respectively) in Ku80ko (Fig 3E). For both genes, transgenic parasites were readily obtained and cloned. Absence of the ACL and ACS ORFs and their replacement by a selection cassette in individual clones was validated by genomic PCR (Fig 3E), thus, supporting the prediction made by the model that these genes are both dispensable for the survival of *T*. *gondii* tachyzoites. No significant defect could be observed in the overall lytic cycle of these knockout mutants as represented by plaque assays performed in human foreskin fibroblast (HFF) monolayers. Indeed, both ACLko and ACSko parasites formed lysis plaques of similar sizes when compared to wild type (Ku80ko) parasites (Fig 3F). Moreover, after 24h of intracellular growth, most vacuoles of the ACLko strain contained 4 to 8 parasites, which is comparable to the number of parasites per vacuole for cells infected with Ku80ko or ACSko (Fig 3G). Finally, growth competition assays between mutants and wt parasites, which would detect mild loss of fitness, showed no significant defect either.

**Fig 4:**
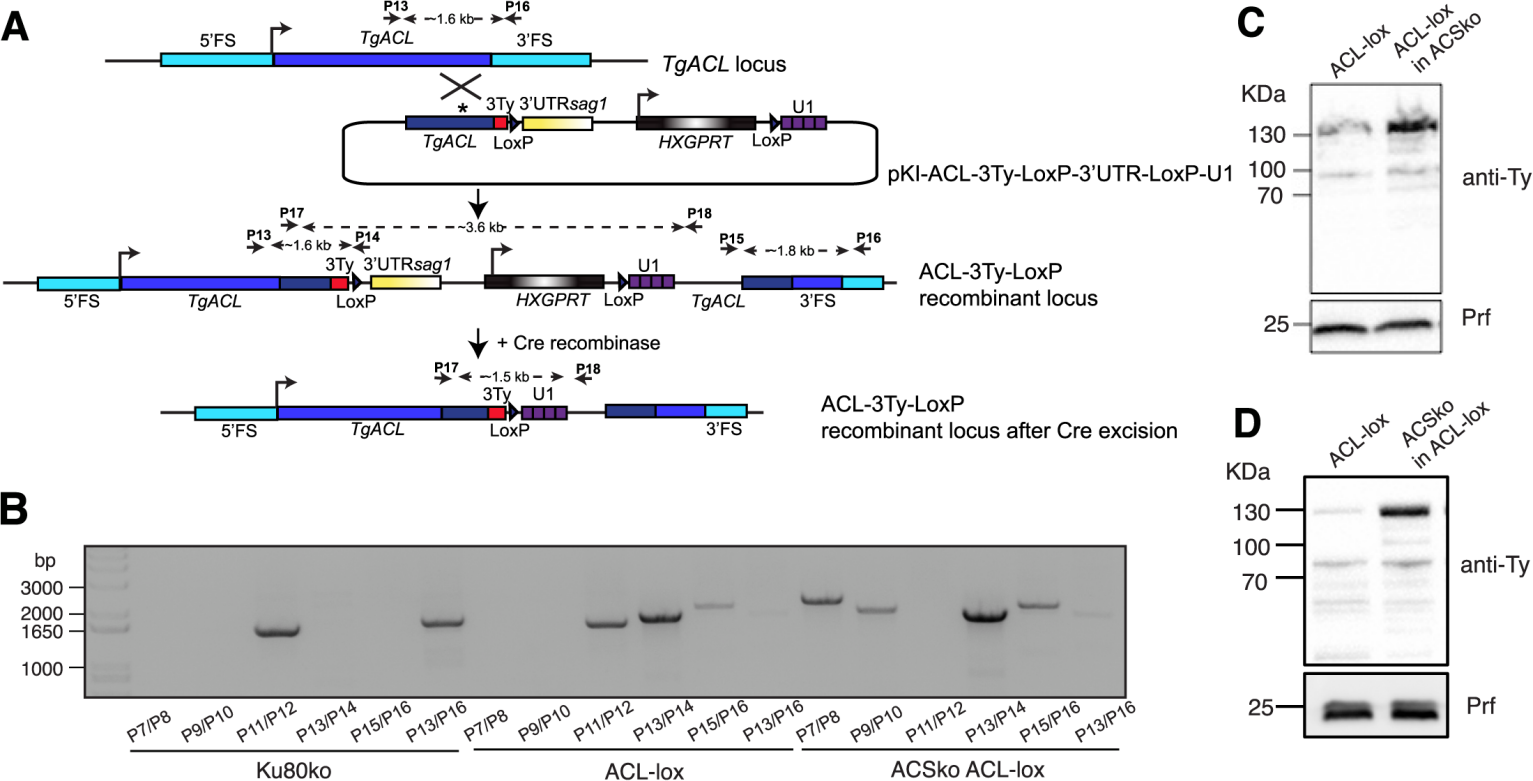
Generation of an inducible ACL knockdown in ACSko parasites. (A) Schematic representation of the U1 snRNP-mediated ACL gene silencing with Cre-recombinase dependent positioning of U1 in Ku80ko wildtype and ACSko parasites. (B) PCRs performed on genomic DNA extracted from Ku80ko, ACL-lox, ACSko/ACL-lox validating integration of the pKI-ACL-3TyLox3’UTRLoxU1 construct to knock down ACL in the different strains. The sequences of the primers can be found in S7 Table (C and D). Immuno-blot of total lysates from ACL-lox, ACSko/ACL-lox where ACL-lox was integrated in the ACSko strain or ACS was knocked out in ACL-lox. Both independent lines show increased levels of ACL when ACS is absent. Western blot was performed using anti-Ty antibodies. Anti-TgProfilin (Prf) represents a loading control.

In order to assess whether a double knockout of ACS and ACL is lethal for *T*. *gondii* as suggested by ToxoNet1, we first attempted to disrupt ACL by single homologous recombination in the middle of the ORF in the ACSko parasite background (same strategy as presented in Fig 3B but leading to a truncation of the protein and removal of the catalytic site). While we were able to interrupt the ACL gene in Ku80ko parasites, we failed to generate such a mutant in the ACSko background. This result strongly suggested that ACS and ACL together are critical for the biosynthesis of cytosolic acetyl-CoA. To further confirm the synthetic lethality between ACS and ACL we generated a conditional knockdown of ACL in Ku80ko and in ACSko by U1 snRNP-mediated gene silencing with Cre-recombinase dependent positioning of U1, as has been recently developed in *T*. *gondii* [41] (generating ACL-lox and ACSko/ACL-lox respectively; Fig 4A). Following Cre-mediated recombination, the endogenous 3’untranslated region is excised and a U1 recognition site is placed adjacent to the termination codon. Consequently the ACL pre-mRNAs are cleaved at the 3’-end and degraded, leading to a highly efficient knockdown of the gene. Loss at the protein level can be assessed by immuno-detection of the C-terminal 3Ty-tag (Fig 4A). Correct integration of the construct was confirmed by genomic PCR (Fig 4B). Importantly, upon deletion of ACS the level of endogenous ACL protein was significantly increased compared to ACL-lox (Fig 4C). This change in level of ACL in the absence of ACS was reproducibly confirmed by generation of a second independent transgenic parasite line where the *ACS* gene was disrupted in the ACL-lox background strain (Fig 4D).

**Fig 5:**
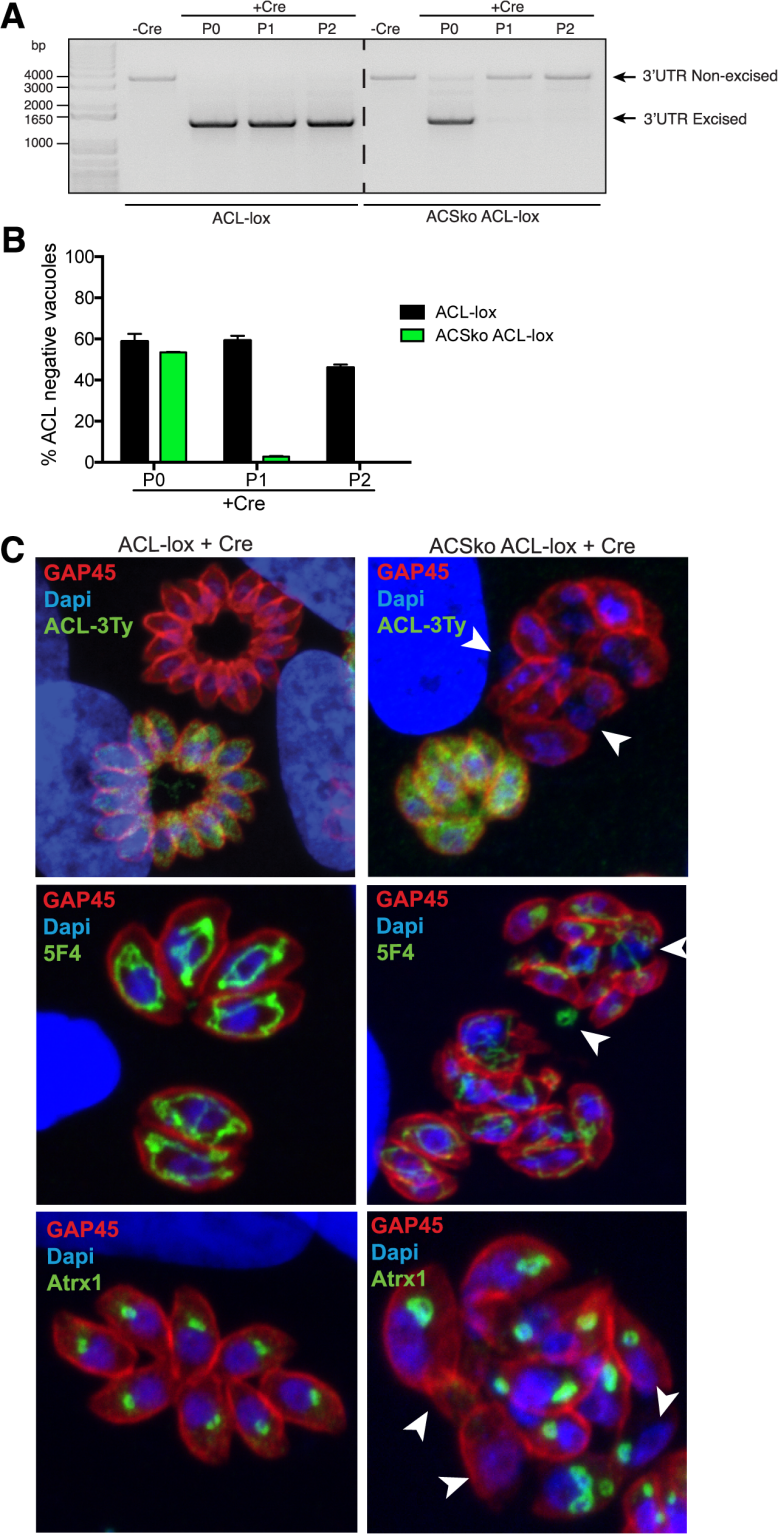
ACS and ACL are dually essential. (A) Following transfection of ACL-lox and ACSko/ACL-lox with Cre recombinase, excision and repositioning of U1 is followed by genomic PCRs over several culture passages (every 48h) using primers P17/P18 as depicted in Fig 4A (P0, extracellular parasites 48h after Cre transfection; P1, extracellular parasites passaged once ~96h post transfection; P2, extracellular parasites passaged twice ~140h post transfection). (B) Histogram showing percentage of excised parasites over several passages (every 48h) in ACL-lox and ACSko/ACL-lox populations following transfection with Cre recombinase. Excised parasites were visualized by IFA looking for loss of ACL-3Ty signal. Due to fluctuation in transfection efficiency, data from one biological replicate is shown and represents mean ± SD from 3 technical replicates. 3 biological replicates were done and gave the same results. (P0, intracellular parasites 30h after Cre transfection; P1, intracellular parasites passaged once ~72h post transfection; P2, intracellular parasites passaged twice ~100h post transfection). (C) Immunofluorescence assay confirms the loss of ACL-3Ty and parasite pellicle integrity in a subset of vacuoles 30h following transfection of ACL-lox and ACSko/ACL-lox strains with a Cre recombinase expressing plasmid. IFAs were stained using anti-Ty, anti-GAP45 (Pellicle), anti-5F4 (mitochondrion) or anti-Atrx1 (apicoplast) antibodies and DAPI (nucleus). Arrowheads highlight nuclear and mitochondrial material lost in the vacuolar space and loss of the apicoplast due to loss of pellicle integrity.

To conditionally disrupt ACL in the ACSko, the ACL-lox and ACSko/ACL-lox parasites were transfected with a plasmid transiently expressing the Cre recombinase. While ACL-lox excised parasites could be readily propagated in culture the ACSko/ACL-lox excised parasites were lost after the first passage as monitored by genomic PCR analyses of the two excised parasite populations. While genomic recombination of excised parasites could be readily detected in the original transfected parasites (P0), the signal was lost immediately after the first passage of these cultures (P1 and P2) (Fig 5A) indicating a rapid deleterious effect. Thirty hours post transfection of Cre recombinase, the loss of ACL-3Ty-tagged protein was evident by IFA in about 50% of the ACL-lox and ACSko/ACL-lox vacuoles. While ACL-lox parasites lacking ACL could be propagated in culture, parasites lacking ACL in the ACSko/ACL-lox strain were immediately lost in the first culture passage (Fig 5B). While ACL-lox excised parasites appeared normal, most vacuoles from excised ACSko/ACL-lox parasites exhibited a severe morphological defect and impairment in the parasite division process with a loss of pellicle integrity as seen by perturbation of GAP45 staining (Fig 5C). Furthermore these parasites appear to continue dividing their nuclei, mitochondrion and apicoplast, but fail to form daughter cells, resulting in organelle accumulation in the vacuolar space (Fig 5C).

To determine whether the deleterious phenotype and loss of pellicle integrity could be attributed to a block in type I fatty acid synthesis following depletion in cytosolic acetyl-CoA, the gene coding for *T*. *gondii* FASI was disrupted in wild type RH strain parasites by CRISPR/Cas9 mediated genome editing [42]. Trangenic parasites were obtained following double-stranded breaks generated by Cas9 at a position downstream of the FASI ATG. Two independent clones were sequenced to confirm the introduction of frame shift mutations (S1A Fig) No defect in the lytic cycle could be observed in these parasites (S1B Fig), which is in accordance with the ToxoNet1 prediction that FASI activity is not essential for parasite survival. Taken together, these data firmly support the predictions made by the model regarding the individual dispensability of ACS and ACL and the synthetic lethality of ACL in the absence of ACS.

## Discussion

ToxoNet1 constitutes a full genome-scale reconstruction of metabolism within *T*. *gondii*, which can simulate growth of the parasite *in silico* and infer essentiality of its genes. It considerably extends the scope of previous work [10] and contributes to better understanding of the limits in the metabolic capabilities of this opportunistic human and animal pathogen. While the modelling efforts of Song et al were aiming to reveal strain-type specific differences in metabolism of *T*. *gondii*, in the present study we reconstructed an independent model that represents the potential scope of the metabolic capabilities of the parasite independently of the life-stage and strain-type. Aiming at the most reliable gene essentiality predictions we used many alternative assumptions on the range of accessible substrates and transportability of metabolites. We have also achieved more comprehensive coverage of all the metabolic capabilities of the parasite and implemented functional gene-protein-reaction associations enabling rigorous gene deletions studies. Hereafter we provide a more in depth discussion on the key aspects of our approach.

There are two distinct approaches for model reconstruction process that are commonly referred to as top-down and bottom-up [43]. The top-down approach is usually applied when detailed experimental data about the majority of individual system components is scarce. Conversely, for the bottom-up approach, a significant body of relevant primary literature is a necessary prerequisite. These two approaches are very much complementary and this is why increasingly more studies combine them in order to achieve the best results. This was also the case in the ToxoNet1 reconstruction efforts: the first draft model was a hybrid of the output generated by RAVEN Toolbox and a significantly smaller manually reconstructed (and highly curated) model, which was built upon the similar model of *P*. *falciparum* [44] used as a template. This bottom-up small-scale model allowed capturing of the features that were not identified by functional annotations, such as formation of multi-enzyme complexes for certain enzymatic activities (e.g. the mitochondrial BCKDH complex that carries out the pyruvate dehydrogenase function [27]) as well as cofactor specificities for the enzymes.

Genes with metabolic functions in ToxoNet1 represent a modest fraction (c.a. 9%) of the *T*. *gondii* protein-coding genes, which is comparable but greater than in the genome-scale models of *P*. *falciparum* (7%) [8], *L*. *major* (6.7%) [20] and *Cryptosporidium hominis* (5.5%) [7]. ToxoNet1 provides a broader and more complete coverage of the metabolic capabilities of the parasite with 145 additional enzyme-coding genes (38% greater) compared to the earlier metabolic model of *T*. *gondii* [10]. Comparison of the number of metabolic reactions with the genome-scale models of the malaria parasite *P*. *falciparum* [8,9,13] confirms the common view that *T*. *gondii* possesses broader metabolic capabilities (Table 5). These differences in metabolic capabilities could be partially responsible for the observations that *T*. *gondii* can infect a very broad range of cell types for asexual replication, while *Plasmodium* spp. can only replicate within specific tissues.

**Table 5:** Comparison of the number of metabolites, reactions and genes between the models for *T*. *gondii* and *P*. *falciparum*.

Metabolic models of apicomplexan parasites	Information about the models			
	Metabolites	Reactions	Genes	Compartments
*P*. *falciparum* [9]	850	998	579	c, m, a, er, n, v, g
*P*. *falciparum* [8]	616	656	366	e, c, m, a
*T*. *gondii* [10]	384	400	382	c, m, a, er, im
*T*. *gondii*, this study	1019	1089	527	e, c, m, a

Abbreviations: e—extracellular space, c—cytosol, m—mitochondrion, a—apicoplast, n—nucleus, er—endoplasmic reticulum, g—Golgi complex, v—digestive vacuole, im—mitochondrial intermembrane space.

ToxoNet1 contains confidence estimates (e-values and bit-scores) for each gene annotated as encoding certain metabolic activities. This has allowed us to suggest a number of metabolic functions for genes that did not have annotations with E.C. numbers in the ToxoDB database (S4 Table). For example, TGME49_237140 (an “ethylene-inducible protein” in ToxoDB without an E.C. identifier) was annotated as pyridoxal 5'-phosphate synthase pdxS subunit (E.C. 4.3.3.6) with very high confidence estimates (e-value 5.30x10^-141^ and bit-score 479). Accordingly, an orthologous gene (PF3D7_0621200) is annotated as pyridoxine biosynthesis protein (PDX1) in *P*. *falciparum*, yet without an E.C. number assigned. There was also a set of genes with low confidence in their function, despite their annotation in ToxoDB (e.g. TGME49_305840 had a low sequence identity to known nicotinate-nucleotide adenylyltransferases with an e-value of 4.70x10^-15^ and a bit-score of 60.6). These two examples demonstrate the potential of the RAVEN Toolbox to improve genome annotation and produce models with confidence estimates that enable evaluation and, potentially, future corrections of the existing models.

ToxoNet1 contains 260 unique dead-end metabolites, which currently can be only produced or only consumed in the network. We kept them in the model in order to allow future developments and expansion of the scope of the model. Further definition of the metabolic routes can utilise these dead-end metabolites and contribute to a more complete understanding of the metabolic capabilities of the parasite. For instance, the tRNAs loaded with the respective amino acids were included to enable future extension of the model to the representation of protein synthesis.

We chose to impose relaxed constraints in terms of subcellular compartments due to rather high uncertainty in subcellular localisation of the enzymes as well as largely unknown capabilities of the parasite to transport metabolites across its organellar membranes. We assigned the enzymes with the corresponding reactions to their putative compartments and allowed a broad range of metabolites to be transported across the compartment boundaries. This approach to compartmentalisation ensures minimal bias in our results due to underestimated transport capabilities or incorrect assignment of enzymes to subcellular compartments. We expect subsequent refinements of ToxoNet1 with more stringent compartmentalisation as more reliable, high-throughput computational and experimental methods are becoming available for subcellular localisation of enzymes and functional annotation of transporter proteins in *T*. *gondii*. Our objective of this work is to provide ToxoNet1 as a resource for the community and therefore we avoided imposing constraints and hypotheses, which are not well-tested and confirmed that could contaminate the model and our results.

To understand the nutritional requirements of *T*. *gondii*, we implemented an algorithm that identifies and ranks the minimal number of substrates required for growth in ToxoNet1. The results indicate that uptake of as few as 19 of 230 substrates allows the model to produce all biomass building blocks. Moreover, we identified 2592 sets of 19 substrates that can allow growth, and 10 substrates common to all these sets (Table 3). These 10 substrates are compounds that are not synthesized *de novo* by *T*. *gondii* and are the precursors for biomass building blocks and essential cofactors. Interestingly, the uptake of S-adenosyl-homocysteine provided precursors for multiple biomass building blocks simultaneously (purine nucleotides, threonine, methionine and their derivatives). Among the alternative substrates we identified 7 carbon sources: five hexoses and two pentoses (ribose and deoxyribose), which can be incorporated into the metabolism by a ribokinase enzyme. Uptake of pentoses through a hexose transporter has been reported in the protozoan parasite *L*. *major* and may potentially represent an additional level of versatility in meeting the need of a carbon source, similarly to the previously observed in *T*. *gondii* utilization of glutamine [45,46]. We could envision such need as the parasite uses various host cells, where the set of available substrates may vary between cell types.

Validation of genome-scale models using metabolic tasks is an approach developed to evaluate metabolic capabilities of the models in a systematic manner. To date it has been applied to several models [11,47,48] for testing whether the major metabolic functions can be fulfilled and the biochemical pathways that support these functions were represented correctly. ToxoNet1 is the first metabolic model of an apicomplexan parasite where the reconstruction process involved the validation of metabolic tasks. Using this approach we explored the capability of this pathogen to produce *de novo* biomass building blocks from glucose and inorganic substances. Interestingly, almost all the cofactors of metabolic enzymes, with the exception of pyridoxal phosphate cannot be produced *de novo*. Their synthesis required precursors that contained certain chemical moieties (Table 2). This suggests that virtually all the metabolic functions of the parasite depend on an adequate supply of specific precursors by the infected host cell. Furthermore, we found that *T*. *gondii* requires uptake of almost all the amino acids, which are essential for the host, with the possible exception of threonine (discussed below). Notably, *T*. *gondii* lacks the pathway for arginine synthesis, which can be produced by human cells, however arginine is growth limiting for human cells because its *de novo* synthesis is insufficient and thus supplemental uptake is required [49]. This suggests that during infection and growth of the parasite, the biosynthetic capabilities of the host can be significantly compromised due to competition for essential and growth limiting amino acids and vitamins.

Threonine is the only amino acid essential for human cells and which *T*. *gondii* can potentially produce. However, this pathway includes the enzyme homoserine dehydrogenase (E.C. 1.1.1.3), the encoding gene for which is not identified to date in the genome of the parasite. Data from ToxoDB suggests that the genes encoding the enzymes of the threonine biosynthesis pathway, namely aspartokinase (TGME49_227090), threonine synthase (TGME49_220840), homoserine kinase (TGME49_216640) and aspartate-semialdehyde dehydrogenase (TGME49_205420), are expressed at low levels in the tachyzoite stage. Homoserine kinase has been detected in the proteome of oocysts [50], the infective forms of the parasite that can survive for extensive periods of time outside the host cell [51]. Taken together, these observations indicate that the whole pathway from aspartate to threonine might not be functional in certain life stages and therefore *T*. *gondii* could be a conditional threonine auxotroph. Should threonine biosynthesis also be active in the other life stages it could represent a selective drug target for inhibition of parasite replication without affecting the mammalian host cells. Maintenance of some metabolite pools in *T*. *gondii* may be a result of both uptake and *de novo* production. Hence, if the complete set of genes of a metabolic pathway is present in the genome of the parasite, this pathway is not necessarily active and will not meet the demands of the parasite regarding the products of the pathway. Fox *et al*. showed that salvage of pyrimidine nucleotides from the host cell can support *in vitro* but not *in vivo* survival of *T*. *gondii* mutants with disrupted *de novo* pyrimidine synthesis pathways [52]. Therefore, accurate consideration of metabolism in the host and pathogen together is central for a better understanding of the metabolic needs and capabilities of *T*. *gondii*.

Our study represents the first model-based gene essentiality predictions for *T*. *gondii*. The earlier efforts to infer essentiality of genes in this parasite were reported by Gautam et al. [53]. Their approach for producing the list of putatively essential genes was largely dissimilar to ours and relied on conservation of the enzymes across parasitic and free-living species with further pruning based on the literature data. We believe that our modelling approach, which takes into account many more inputs and constraints, produces more reliable gene essentiality predictions, and it follows the standard modelling procedures established by the community [12,54]. Nevertheless, we consider that the part of the workflow proposed by Gautam et al. can be complementary to our study, which also includes larger number genes with the updated functional annotation we performed here. This future study will prioritise the putatively essential genes according to their apparent utility as drug targets with minimal risk of off-target effects on the host cell metabolism.

Using ToxoNet1 we predicted a minimal set of 53 enzyme-encoding genes to be indispensable for parasite replication within human cells. The majority of these genes (49 out of 53) have orthologs in the malaria parasite *P*. *falciparum* and 32 of them were predicted as essential in the existing metabolic models [38]. Evidence of essentiality for metabolic enzymes in *T*. *gondii* is currently scarce, thus, we could not test the majority of our essentiality predictions (Table 5). However, new technologies such as CRISPR/Cas9-mediated genome editing, hold promise of forthcoming high-throughput gene knockout studies in various organisms including *T*. *gondii* [42]. The dataset of experimentally established gene essentiality will provide an important validation of ToxoNet1 and a prerequisite for its further refinement and expansion.

We predicted synthetic essentiality of 20 pairs of genes, which represent two distinct cases. In the first case the pair of genes encode two isoenzymes that catalyse the same reaction in the same compartment. For example, the genes TGME49_318580 and TGME49_285980 encode isoenzymes, which catalyse the phosphoglucomutase reaction (E.C. 5.4.2.2) and both were experimentally localised to the cytosol [33]. This reaction produces glucose-1-phophate in the cytosol, which is an indispensable precursor for starch and nucleotide-sugar metabolism. Therefore, simultaneous deletion of these two genes is a synthetic lethal. In the second case of synthetic essentiality each gene encodes for an enzyme of a different reaction but with a common product, which is necessary for biomass synthesis. Thus, the two different enzymes can substitute for each other (sometimes indirectly) for production of an essential metabolite. For example, we established experimentally the synthetic lethality between the genes encoding acetyl-CoA synthase (ACS) and ATP-citrate lyase (ACL), both of which produce acetyl-CoA in the cytosol of the parasite. This central metabolite participates notably in fatty acid synthesis, fatty acid chain elongation and in acetylation of proteins. We were able to rule out experimentally that the severe phenotype observed upon depletion of cytosolic acetyl-CoA was due to an impact on FASI since parasites lacking FASI did not exhibit any significant impairment in the lytic cycle.

Moreover given that deletion of the genes coding for elongases previously reported [55] did not phenocopy the lethality observed when attempting to delete ACS and ACL simultaneously, we suspect that blockage of protein acylation might be responsible for the severe consequences of deletion of cytosolic acetyl-CoA. In this context, we experimentally confirmed that the *T*. *gondii* acetyl-CoA transporter (AT1) is localized in the membrane of the ER and is anticipated to deliver acetyl-CoA to the secretory pathway. It would be interesting to examine the importance AT1 for parasite survival. In contrast, the mitochondrion and the apicoplast should not be affected since these compartments have their own routes for production of acetyl-CoA: (i) in the apicoplast it is produced by the pyruvate dehydrogenase (PDH) complex [56], and (ii) in the mitochondrion synthesis of acetyl-CoA is carried out by the BCKDH complex [27]. The predicted synthetic lethality between ACS and ACL suggests that these are the only two significant sources of acetyl-CoA in the cytosol, which was not assessed by previous experimental studies and is formally demonstrated here.

Furthermore this synthetic lethality suggests that there is no, or no significant, transport of acetyl-CoA to the cytosol from the mitochondrion or the apicoplast. This further confirms that, unlike acetate, acetoacetate could not be a source of cytosolic acetyl-CoA and therefore the metabolic role of acetoacetyl-CoA, considered to be a dead-end metabolite in ToxoNet1, requires further investigation. Unexpectedly, the deletion of the gene coding for ACS leads to a reproducible and immediate increase in abundance of ACL protein, a possible compensatory adaptation that has not been reported previously. It is unclear whether the capacity of the parasite to respond to the absence of ACS results from an increase at the level of transcription or protein stability but this intriguing mechanism of adaptation deserves further investigation. ToxoNet1 can assist in comprehensively embracing the various routes that *T*. *gondii* employs to produce acetyl-CoA in different subcellular compartments.

Beyond this study ToxoNet1 can be used as a global metabolic context for integration and interpretation of various high-throughput experimental data, similarly to the studies made in *P*. *falciparum* and other eukaryotic pathogens [38]. Of particular interest is the integration of experimental data collected on different life stages of *T*. *gondii* that will allow the model to yield context-specific predictions and, potentially, reveal valid drug targets as well as fundamental knowledge regarding the stages implicated in persistence and transmission of this important human and animal pathogen.

## Materials and Methods

### Draft reconstruction of ToxoNet1

The reconstruction process started with generation of a draft metabolic network of *T*. *gondii* based on the annotation of its protein sequences as extracted from the ToxoDB database [57]. Within the framework of the RAVEN Toolbox these protein sequences were compared to the hidden Markov models (HMM) generated for each KEGG [17] orthology group [11]. In the cases when e-values for the matches between a *T*. *gondii* protein and an HMM were smaller than the specified e-value cutoff (10^–20^), the enzymatic reactions associated with the corresponding KEGG orthology ID were added to the draft metabolic network. As a result we have obtained a set of metabolic reactions linked to genetic loci of *T*. *gondii*. At this stage the model did not contain information about either subcellular compartments, or about transport of metabolites. We next merged this model with a manually curated, small-scale metabolic reconstruction that we previously built based on the *P*. *falciparum* model [44].

Similarly to Huthmatcher *et al* [9]., we removed reactions with generic metabolites (such as “protein”, “dNTP”) and replaced non-unique metabolite identifiers with unique ones when they corresponded to the biochemically equivalent entities (e.g. (S)-Lactate and L-lactate were replaced with L-lactate).

### Compartmentalization

The parasitophorous vacuole (PV) space, which secludes *T*. *gondii* from the host cell cytosol, is the outermost compartment represented in ToxoNet1. Extracellular compartment of the model corresponds to the PV space. A eukaryotic organization of intracellular space of the parasite also includes a number of compartments, among which we chose to represent in ToxoNet1 the most relevant to metabolism, namely: mitochondrion, apicoplast and cytosol. The following sequence-based localization predictors were used to suggest putative subcellular localization of the enzymes: TargetP [16] (version 1.1), MitoProt II [15] (version 1.101) and ApicoAP [14] (version 1)). As an input data we used sequences of ORFs extracted from ToxoDB (v.9, strain ME49) for the genes included in ToxoNet1. Computational localization predictions were reconciled with the literature evidence for *T*. *gondii* and *P*. *falciparum* in the cases when the latter were available (see S1 Table).

Transport reactions were included to link the majority of the metabolites in non-cytosolic intracellular compartments with their cytosolic counterparts; such transports were not created for phosphorylated metabolites or those that contained [acyl-carrier protein] ([ACP]) or Coenzyme A (CoA) moiety attached (with the exception of the apicoplast-to-cytosol transport of IPP, and DMAPP as well as the mitochondrion-to-cytosol transport of ATP).

### Metabolic tasks

Testing metabolic tasks is a built-in functionality of the RAVEN Toolbox [11] meant for verification of correct metabolic capabilities in genome-scale models. Formulation of this function allows one to test production of a certain metabolite(s) (or flux through particular reactions) with strictly specified inlet and outlet of metabolites. For instance, a metabolic task “*de novo* synthesis of ATP” was formulated as following: given unlimited uptake of glucose, oxygen, inorganic phosphate, sulfate and ammonium, can the model produce ATP. The model failed to accomplish this task, which is consistent with the literature knowledge about auxotrophy of *T*. *gondii* for purines [58]. Thus, the task could be passed only when we added to the list of available compounds hypoxanthine, adenine or another molecule containing a purine moiety. In a similar manner we have tested over a 60 metabolic tasks (all in S2 Table) to ensure realistic metabolic capabilities of the model.

### The range of available substrates

We simulated metabolism of the host cell using the most recent tissue-unspecific model of human metabolism [19]. The Recon2 model was modified in terms of available substrates to reflect growth on the defined minimal medium (Dulbecco's Modified Eagle's Medium with glutamine and glucose). The PV which secludes the parasite from the host cell cytosol had been reported as being permeable to small-molecule metabolites with molecular weights below 1500 Da [59] and thus imposes no relevant constraint to the metabolites we considered in ToxoNet1. Thus, all the molecules that could be produced from the medium components in the cytosol of the host cell were assumed to be potentially accessible for the parasite provided that they satisfy the following criteria: host-supplied substrates were only small molecules (below 1.5 kDa) that were not phosphorylated or bound to-CoA,-[ACP] or carnitine. We also assumed that the parasite could potentially dispose of a wide range of metabolic by-products from its cytosol into the host cell. Thus, we added sink reactions for the same 230 metabolites that were assumed to be host-supplied. As an exception we also allowed direct uptake into the cytosol of the following five generic metabolites: “reduced acceptor”, “sulfur donor”, “acyl-CoA”, “carboxylate” and “1-acylglycerol”; two non-generic metabolites: selenite and myo-inositol, and the appearance of apoprotein in the mitochondrion and the apicoplast.

### Biomass composition

In order to simulate replication of the parasite and assess the essentiality of genes, reactions and substrates, we assumed maximisation of flux through the biomass reaction to be the objective function of ToxoNet1. It represents cellular replication as a reaction that consumes pre-defined amounts of metabolites defined as small-molecule biomass precursors as well as energy in the form of ATP. We used the biomass reaction from the previous study [10] as a template and introduced the following modifications: we extended this biomass reaction with the following cofactors—NAD, NADP, FAD and lipoylated protein necessary for pyruvate dehydrogenase (PDH) activity in the mitochondrion and the apicoplast; we changed the compartment for lipid precursors from endoplasmic reticulum (in Song et al. [10]) to the cytosol (no ER compartment in ToxoNet1), and for L-lysine from mitochondrial to cytosol. The presence of the mitochondrial pathway for L-lysine production remained obscure, thus in ToxoNet1 it is acquired from the host instead of gap-filled *de novo* production in the parasite and sequestered towards biomass from the cytosol.

### Flux balance analysis (FBA) and substrate dispensability simulation

FBA is a standard computational approach for exploration of the metabolic capabilities represented in constraint-based models; principles, computational implementation of FBA as well as the key assumptions are extensively described elsewhere [60].

In the absence of clear knowledge on the scope of substrates that the parasite can take up from the host we made the following assumption: all the metabolites present in the host cell cytosol are potentially accessible for the parasite except those that are phosphorylated, bound to coenzyme A, acyl-carrier protein or carnitine. A list of molecules that satisfy these criteria was generated using the recent tissue-unspecific model of human metabolism Recon2 [19] (the list of the substrates is in S6 Table).

Minimal sets of metabolites necessary for the production of biomass were explored using an in-house developed mixed-integer linear programming algorithm. For each of the exchange reactions that allowed uptake of a substrate into the model (i.e. 230 host-supplied metabolites) we added one binary variable that denoted its utilization. Our algorithm solved the model subject to minimization of the sum of the binary variables thus yielding a minimal set of uptakes that allowed the model to simulate growth. After each iteration the algorithm added one new constraint to the model to assure that the following set would include at least one different uptake reaction compared to all the previously generated ones. The iterations were repeated until no more alternative sets of the same length could be found.

### Essentiality studies

We performed simulation of gene deletion using a conventional approach [61] that implies evaluation of gene-reaction associations that include the gene of interest, preventing flux through corresponding metabolic reactions and an attempt to achieve a doubling time of 4.5 hours (specific growth rate of 0.15 h^-1^). Similar approaches were used for double gene deletion studies—simultaneously blocking reactions associated with all pairs of genes that were not predicted as singularly essential. In reaction deletion simulations we blocked flux through every single reaction in ToxoNet1 one at a time. Subsequent attempts at achieving the wild-type doubling time in the absence of the reaction indicated whether the gene was dispensable or not.

### *T*. *gondii* strains and culture

*T*. *gondii* tachyzoites (RH*ku80-ko* (Ku80ko), RH*ku80-ko/ACS-ko* (ACSko), RH*ku80-ko/ACL-ko* (ACLko), RH*ku80-ko*/ACL3Ty-LoxP3’UTRLoxP-U1 (ACL-lox), RH*ku80-ko*/*ACS-ko*/ACL3Ty-LoxP3’UTRLoxP-U1 (ACSko/ACL-lox), RH/*FASIko* (FASIko)) were grown in confluent Human Foreskin Fibroblasts (HFF) and maintained in Dulbecco’s Modified Eagle’s Medium (DMEM, Life technology, Invitrogen) supplemented with 5% foetal calf serum, 2 mM glutamine and 25 μg/ml gentamicin in a humidified incubator at 37°C with 5% CO_2_.

### Cloning of DNA constructs

All amplifications were performed with LA Taq (TaKaRa) polymerase and primers used are listed in S7 Table.

*Knock-in of ACS*: *(pKI-ACS-3Ty)* The genomic fragment of ACS (TGME49_266640) was amplified using primers ACS-1 and ACS-2 prior to digestion with with *KpnI* and *SbfI* and subsequent cloning in the same sites of pTub8MIC13-3Ty-HXGPRT [62] to introduce 3 Ty-tags at the C-terminus of the endogenous locus. Before transfection pKI-ACS-3Ty was linearized with *SnaBI*.

*Knock-in of AT1*: *(pKI-AT1-3Ty)* The genomic fragment of AT-1 (TGME49_215940) was amplified using primers AT1-1 and AT1-2 prior to digestion with *KpnI* and *NsiI* and subsequent cloning in the same sites of pTub8MIC13-3Ty-HXGPRT [62] to introduce 3 Ty-tags at the C-terminus of the endogenous locus. Before transfection pKI-AT1-3Ty was linearized with *HindIII*.

*Knock-in and knock-down of ACL*: *(pKI-ACL-3Ty and pKI-ACL-3Ty-LoxP-3’UTR-LoxP-U1)* Genomic fragment of ACL (TGME49_223840) was amplified using primers ACL-1/ACL-2 and subsequently digested with *KpnI* and *NsiI* prior to cloning in the same sites of the pTub8MIC13-3Ty-HXGPRT for 3Ty-tag knock-in [62], or the modified C-terminal destabilization vector pG152-3Ty-LoxP-3’UTRSag1-HXGPRT-LoxP-U1 [41]. Prior to transfection both plasmids were linearized with *XhoI*.

*Knockout of ACS*: *(pTub-CAT-ACS-ko)* around 1.5kb of the 5’ and 3’ flanking regions of ACS were amplified using primers ACS-3/ACS-4 and ACS-5/ACS-6 respectively. The 5’ flanking region was then cloned between *KpnI* and *XhoI* restriction sites of the pTub5-CAT and the 3’ flanking region between the *BamHI* and *NotI* sites. The plasmid was cut with *KpnI* and *NotI* prior to transfection.

*Knockout of ACL*: *(pTub-HXGPRT-ACL-ko)* around 1.5kb of the 5’ and 3’ flanking regions of *ACL* were amplified using primers ACL-3/ACL-4 and ACL-5/ACL-6 respectively. The 5’ flanking region was then cloned between the *KpnI* and *XhoI* sites of the p2855-HXGPRT plasmid and the 3’ flanking region between the *BamHI* and *NotI* sites. The plasmid was cut with *KpnI* and *NotI* restriction enzymes prior to transfection.

*Frameshift knockout of FASI using CRISPR/CAS9 plasmid [42]*: This vector has been generated using the Q5 site-directed mutagenesis kit (New England Biolabs) with the vector pSAG1::CAS9-U6::sgUPRT as template (a gift from Dr. L.D. Sibley). The UPRT-targeting gRNA was replaced by the FASI (TGME49_294820) specific gRNA using the primer pairs gRNA-FASI/gRNA-rev (gRNA highlighted in red in S7 Table).

### Parasite transfection and selection of stable transformants

Parasite transfections were performed by electroporation as previously described [63].The *hxgprt* gene was used as a positive selectable marker in the presence of mycophenolic acid (25 μg/mL) and xanthine (50 μg/mL) for pKI-ACS-3Ty, pKI-AT1-3Ty, pKI-ACL-3Ty, pKI-ACL-3Ty-LoxP-3’UTR-LoxP-U1 and pTub-HXGPRT-ACL-ko vectors transfected in Ku80ko or ACSko, as previously described [64].

Ku80ko was transfected with pTub-CAT-ACS-ko and 20μM chloramphenicol was used to select resistant parasites.

Resistant parasites were cloned by limiting dilution in 96 well plates and clones were assessed by genomic PCR.

To efficiently disrupt the *FASI* locus, 30 ug of the *FASI* gRNA-specific CRISPR/CAS9 vector was transfected into wild type RH parasites. 48 hours after transfection, GFP positive parasites were sorted by flow cytometry and cloned into 96-well plates using a Moflo Astrios (Beckman Coulter). Individual clones were then analysed by sequencing.

### Preparation of *T*. *gondii* genomic DNA

Genomic DNA was prepared from tachyzoites using the Wizard SV genomic DNA purification system (Promega). Correct integration of the different constructs into the genome of the various strains was determined by genomic PCR using GoTaq Green Master Mix (Promega).

### Antibodies

The antibodies used in this study were described previously as follows: polyclonal rabbit anti-GAP45, rabbit anti-TgProfilin [65], monoclonal mouse anti-Ty (BB2), mouse monoclonal anti-F1-ATPase beta subunit (P. Bradley, unpublished) (5F4), mouse monoclonal anti-ATrx1 11G8 [66]. For Western blot analyses, secondary peroxidase conjugated goat anti-rabbit or mouse antibodies (Molecular Probes) were used. For immunofluorescence analyses, the secondary antibodies Alexa Fluor 488 and Alexa Fluor 594-conjugated goat anti-mouse or rabbit antibodies (Molecular Probes) were used.

### Immunofluorescence assay (IFA) and confocal microscopy

Parasite-infected HFF cells seeded on cover slips were fixed with 4% paraformaldehyde/0.05% glutaraldehyde (PFA/Glu) in PBS. Fixed cells were then processed as previously described [67]. Confocal images were generated with a Zeiss (LSM700, objective apochromat 63x/1.4 oil) laser scanning confocal microscope at the Bioimaging core facility of the Faculty of Medicine, University of Geneva. Stacks of sections were processed with ImageJ and projected using the maximum projection tool.

### Western blot analyses

Parasites were lysed in PBS-1% Triton X-100 and mixed with SDS–PAGE loading buffer under reducing conditions. The suspension was subjected to sonication on ice. SDS-PAGE was performed using standard methods. Separated proteins were transferred to nitrocellulose membranes and probed with appropriate antibodies in 5% non-fat milk in PBS-0.05% Tween20. Bound secondary peroxidase conjugated antibodies were visualized using the SuperSignal (Pierce).

### Phenotypic analyses

*Plaque assays*: HFF monolayers were infected with parasites and let to develop for 7 days before fixation with PFA/Glu and Crystal Violet staining to visualize plaques.

*Intracellular growth assays*: HFFs were inoculated with parasites, washed 4h post infection and coverslips were fixed at 24 h post-infection with 4% PFA/Glu and stained by IFA with rabbit anti-TgGAP45. Number of parasites per vacuole was counted in triplicate for each condition (n = 3). More than 200 vacuoles were counted per replicate.

### Software and databases

Flux-balance analysis was performed using MATLAB (Version R2012b, The MathWorks) with CPLEX (ILOG IBM, version 12.51) and Mosek (version 7) solvers; RAVEN Toolbox was used within Matlab environment (version 1.07, downloaded from http://129.16.106.142/tools.php?c=raven). Input data was taken from KEGG database (www.kegg.jp, up to date as of 18/11/2013), ToxoDB (www.toxodb.org, version 9), ApiLoc database (http://apiloc.biochem.unimelb.edu.au/apiloc/apiloc, version 3), and LLAMP portal (www.llamp.net, no evident version tracking).

## Supporting Information

S1 Fig Sequencing from translational start (ATG) of 2 independent clones confirms disruption of the FASI ORF induced by CAS9. (A) PCR for sequencing was done using primers FAS-1/FAS-2 (S7 Table). The gRNA used is written in red. (B) Plaque assays performed with RH, FASIko clone 8 and 10 parasite lines and fixed after 7 days. No significant defect in the lytic cycle could be observed.
(EPS)

S1 Table Subcellular localization of enzymes in ToxoNet1. (XLSX)

S2 Table Metabolic tasks performed on the metabolic network of *T*. *gondii*. (XLSX)

S3 Table Gap-filling reactions introduced in the model and their description. (XLSX)

S4 Table New putative functional annotation for the genes not annotated with E.C. identifiers in ToxoDB. (XLSX)

S5 Table Essentiality predictions (genes, gene pairs, reactions). (XLSX)

S6 Table The list of 230 metabolites assumed as host-supplied substrates in ToxoNet1. (XLSX)

S7 Table Oligonucleotide primers used in this study for cloning and PCR analyses. (PDF)
