# Supplementary material for: Metabolic Needs and Capabilities of Toxoplasma gondii through Combined Computational and Experimental Analysis
Source: PLoS Comput Biol. 2015 May 22;11(5):e1004261. doi: 10.1371/journal.pcbi.1004261 (PMC4441489; doi:10.1371/journal.pcbi.1004261)
Supplement: S7 Table — (PDF) [file pcbi.1004261.s008.pdf]

Table E7. Oligonucleotide primers used in this study for cloning and PCR analyses.

F : Forward, R : Reverse

Cloning :	
Name	Sequence 5'→3' (restriction site underlined)
ACS-1F	GCCGGTACCATGAGCTAAAGCGTGTCGTGC
ACS-2R	GCCCCTGCAGGAGCTTTCGCAAGAGAGCC
ACS-3F	GCCGGTACCCTCAGTCACACACCCTTCTCTC
ACS-4R	GCCCTCGAGCAGGTACGAACGCTGTTAAGATG
ACS-5F	CCGGGATCCGCCGTCGTCGACTTCTATATTTTCTTC
ACS-6R	CCGGCGGCCGCCACGGATCTTGAATTTGTCGGATGC
ACL-1F	GCCGGTACCGAGTACGAAGTGGTCGAAG
ACL-2R	GCCATGCATCCTGCTGGTTCTCTGCGCTG
ACL-3F	CCGGGTACCGAACAAGACTCGTCCCTCG
ACL-4R	CCGACTCGAGAGACGTCTGCAACGGGTG
ACL-5F	CCGGGATCCGCGTCGTTTAAGAGCAAACGAAGC
ACL-6R	CCGGCGGCCGCAAATACATACATACGTAGGTGCC
AT1-1F	CCGGGTACCCTGGGACTCCGTCGAGGTTGTTC
AT1-2R	CCGATGCATCCTTCTCCACTCTCTTCAGCTCGGTTC

PCR analyses		
Figure	Name	Sequence 5'→3'
Figure 3E	P1-ACL	CCGACTTCAGCACAGTCAAC
	P2-HXGPRT	CCGTAGTCTTCAATGGGTTTGGACGC
	P3-HXGPRT	CAGTGACACCGCGGTGGAGG
	P4-ACL	GGCGGACGGCTGTTAATATACG
	P5-ACL	CTTCACAAACGTGGCGGACACG
	P6-ACL	GCAGCGGCCTCTTGAACCA
	P7-ACS	GCTCTACTGCAGGTTGAAG
	P8-CAT	ACACAAGGTGATTGTGTAACACCG
	P9-CAT	CCGTCGACACACGCGCCACATGGG
	P10-ACS	GACTTGTGTAGATGCAAGCAGC
	P11-ACS	CCGGGTACCGGGAGAAGAACAGATCTTTGCAGG
	P12-ACS	CCGTCTCGAGCCTACATTCCGAGGAAACGGTCTG
Figure 4A and 4B	P13-ACL	TACTTCCAATCCAATTTAATGCGCGACTGTATCGGCCAGGGAGCG
	P14-UTRSAg1	GTGACACCTGCAAGCCACAGCGG
	P15-U1	CCGACTAGTATGTGCTGCAAGGCGATTAAGTTGGG
	P16-ACL	CCAGACTGCGACAGAAAC
Figure 5A	P17-ACL	GCCGGTACCGAGTACGAAGTGGTCGAAG
	P18-M13F	GTAAAACGACGGCCAGT
